# The impact of sustainable development on the relevance of accounting information and financial activities: Evidence from Thailand

**DOI:** 10.1371/journal.pone.0313299

**Published:** 2024-11-25

**Authors:** Mongkhol Moolkham

**Affiliations:** School of Interdisciplinary Studies, Mahidol University, Kanchanaburi, Thailand; University of Bologna, ITALY

## Abstract

This study investigates the impact of sustainable development on the relevance of accounting information and financial activities of companies listed on the Stock Exchange of Thailand (SET). The results reveal that earnings per share and book value per share have a positive effect on market value, implying that higher earnings signal strong financial performance, thereby attracting more investor interest. Short-term and long-term debt financing have a negative effect on market value, suggesting that debt financing leads to increased financial risk. Current asset and fixed asset investments have a positive effect on market value by signaling confidence in operational performance. Dividend payouts have a positive effect on market value, demonstrating a commitment to returning value to investors, resulting in a stronger firm reputation and investor perception. However, firms that adhere to sustainable development guidelines face more complex dynamics. The results show that both earnings per share and book value per share have a negative effect on market value, suggesting that while they report high earnings per share and book value per share, these financial metrics cannot alleviate investor skepticism regarding sustainability as a cost of the firm. Short-term debt financing has a positive effect on market value because it provides a flexible and efficient way to fund sustainable investments without diluting equity or incurring long-term debt obligations, while the implications of long-term debt financing and current asset investments are insignificant. Furthermore, the significant positive effect of fixed asset investment underscores the potential long-term benefits of sustainability, despite high initial costs. Lastly, the non-significant negative impact of dividend payouts on market value suggests that the overall effect may also depend on various factors. These results support the idea of efficient market theory, which posits that investors may have negative reactions to what they perceive as financial burdens, diminishing the importance of positive financial metrics and altering market value. This study recommends that policymakers should carefully design regulations and incentives to support sustainable investments. Such approaches may include establishing specific funds, tax incentives, subsidies, and soft loans. Additionally, policymakers need to promote transparency and consistent reporting on the long-term financial benefits of sustainability, which can help reduce investor skepticism and foster a more positive market response. Finally, firms should clearly communicate their long-term sustainability efforts and benefits to investors and various stakeholders, leading to a positive interpretation of the firm’s commitment to sustainable development.

## Introduction

This study is motivated by empirical research by Ohlson [1], which examined the relevance of accounting information, as well as the studies of other researchers such as Dimitropoulos and Asteriou [2], Shamki [3], Mostafa [4], Ahmadi and Bouri [5], and Čupić, Todorović [6]. Value relevance is a critical attribute of accounting information that significantly influences decision-making processes. An increase in the relevance of accounting information is vital for attracting investment, facilitating the sale of established firms’ equities, and enhancing investors’ decision-making capabilities [7]. From investor perspectives, accounting information plays a crucial role in assessing firm performance, acting as a bridge between the management and the information users, providing insights into financial health that are critical to making investment decisions. Similarly, firm financial activities play a crucial role in propelling firm growth because they demonstrate the company’s capacity to effectively manage resources and generate value for its stakeholders. For instance, debt financing, which is crucial for investment expansion, can stimulate earnings growth, resulting in enhanced shareholder value. However, prudent debt management is necessary to mitigate long-term financial risks. Achieving an optimal debt level not only fosters profitability and firm value but also bolsters competitiveness in the business environment [8]. Assets, representing economic resources expected to benefit the firm, play a critical role in optimizing operational efficiency and generating sustainable returns [9]. Asset investment decisions are crucial for efficiently allocating resources, optimizing operations, and generating future returns. Dividend payouts can signal information to investors about future performance [10]. However, excessive dividend payout can adversely affect firm wealth. Balancing debt financing, asset investment, and dividend payouts is a critical strategy for firms aiming to sustain market competitiveness and maximize shareholder wealth. Understanding the implications of these financial activities is crucial for stakeholders, regulators, and researchers to comprehend corporate financial strategies and their effects on market dynamics. These elements have an impact on investors and are often integrated into the investment decision-making process alongside evaluations of firm profitability through key accounting information metrics, such as earnings per share and book value per share. However, empirical challenges remain regarding the impact of accounting information on stock prices in emerging and developing markets, an area that has not been extensively studied [11].

In recent years, there has been a renewed interest in creating value for stakeholders in various contexts [12], closely linked to the integration of sustainable development practices—a crucial strategy for firms that are aimed not at delivering only financial returns but also need to contribute to environmental preservation, foster social inclusivity, and uphold ethical governance standards. This change reflects a broader recognition that sustainable development practices are becoming integral to long-term value creation and essential for maintaining a competitive advantage and meeting stakeholder expectations that are expected to balance traditional financial objectives with broader social and environmental goals and offer several benefits, such as enhancing brand reputation [13], improving employee productivity, increasing operational efficiency, and strengthening relationships with regulators, society, and various stakeholders [14]. These advantages can help firms sustain their market positions over the long term and create opportunities for superior investment projects [15]. However, sustainable development commitments can sometimes lead to overinvestment and other actions that may not align with shareholder interests. Management might undertake socially responsible initiatives that benefit broader stakeholder groups but come at the expense of shareholders, potentially eroding firm value if these initiatives do not generate profitable returns [16]. This raises the question: how do companies committed to sustainable development alter the traditional relationship between accounting information, financial activities, and the market value of firms in emerging markets, particularly in Thailand? While past studies have clarified the impact of accounting information and financial activities on market value, the moderating effect of sustainable development necessitates a reevaluation of how sustainability initiatives interact with accounting information and financial activities. Due to the diverse perspectives of investors on sustainability projects, firms must effectively communicate their commitment to these goals while maintaining financial health. Therefore, the relevance of accounting information must be adapted to reflect not only financial performance but also its sustainability efforts and their implications for future growth and investor confidence. Consequently, this study underscores the importance of integrating sustainability into financial analysis to provide a comprehensive view of corporate performance and value creation in today’s dynamic market environment.

The study of how sustainable development moderates the relevance of accounting information and financial activities to market value is essential for understanding how firms can balance financial performance with sustainability efforts. As investors and stakeholders increasingly prioritize sustainability, which can affect market value too, this study will explore how sustainability practices interact with traditional financial metrics—such as earnings per share, book value per share, debt financing, asset investment, and dividend payouts. Through these examinations, this research illuminates the effect of sustainability on financial decision-making and highlights the necessity to reassess conventional financial metrics as firms incorporate sustainability into their strategies, thereby illustrating its influence on corporate behavior, capital allocation, and investor expectations, particularly in emerging markets. This research contributes to the broader discussion about aligning financial performance with corporate sustainable development and provides a foundation for further exploration of sustainable finance in global markets.

## Literature review and hypotheses

This section provides a comprehensive analysis of its correlation with the variables in this research, as well as a review of past studies related to this subject.

### Accounting information and market value

Accounting information consists of several key metrics, such as earnings per share and book value per share, vital indicators for evaluating financial performance. Earnings per share, calculated by dividing net profits by the number of outstanding shares, indicate firm profitability, while book value per share reflects the portion of firm equity that is attributed to each share of common stock if the firm gets liquidated, calculated by dividing shareholders’ equity by the number of outstanding shares. Both metrics serve as crucial benchmarks for investor decision-making, evaluating a firm’s ability to generate income and create value for its shareholders. The transition from earnings to book value forms the foundation of firm value evaluation [3], and increased transparency in financial information has been shown to positively impact shares, reducing the risk of share collapse [17]. According to the efficient market theory [18], stock markets respond quickly to news. If a firm reports higher profits, the market reacts positively. Conversely, lower profits lead to a negative response, resulting in a decrease in the firm’s market value.

The studies in emerging markets reveal the significant role of accounting information in explaining stock prices. In Egypt, earnings and book values hold substantial explanatory power for stock prices, with earnings demonstrating a stronger influence [4]. Similarly, in Tunisian banks and financial institutions, a significant correlation exists between earnings and shareholders’ equity book value [5]. In Ghana, earnings play a more prominent role in share price fluctuations compared to book value [19], while in Jordan, various accounting metrics positively and significantly impact market value per share [20]. In the MENA region, accounting information is highly relevant to market value, reflecting a strong positive relationship [21]. However, findings from the Amman Stock Exchange suggest that earnings per share, book value per share, dividend payout ratio, and bank size do not influence stock returns [22]. In Serbia, accounting earnings are more value relevant than cash flows, with value relevance increasing after capital market regulatory improvements [6]. In Nigeria, earnings per share, net book value per share, and price-earnings ratio positively influence share prices, while return on equity shows no significant relationship [23]. However, the COVID-19 pandemic has reduced the usefulness of accounting information for investor decision-making in Nigeria, with a decline in the explanatory power of earnings and book value in the post-pandemic period [24].

Studies conducted in the Asian market show that book value per share and net cash flow from investing activities in the NSE Energy Index consistently explain market price variations, whereas profit after tax fails to do so [25]. In Vietnam, earnings and book value of equity positively and significantly influence stock prices, with earnings explaining more variation in stock market values than book value. However, the study notes a declining trend in value relevance from 2010 to 2020, with the 2014 accounting reforms failing to enhance it [26]. In China, the value relevance of financial variables varies, with some increasing and others decreasing, indicating that accounting figures play a role in stock pricing but at different levels. Smaller firms, those with lower growth rates and higher asset tangibility, show more pronounced improvements in value relevance [27]. In Pakistan, changes in earnings are found to be more value-relevant than earnings, reflecting investor priorities [28]. For ASEAN banks in Indonesia, Malaysia, Singapore, Thailand, and the Philippines, both earnings and book value have a statistically positive impact on stock prices, with revenue being more closely linked to value than other variables [29]. In Malaysia, earnings, book value of equity, and cash flow from operations are found to be valuable, and real earnings management moderates their relevance [30]. On the Stock Exchange of Thailand, net income exhibits the highest value relevance across most industries, while property, construction, and resources sectors show comprehensive income as the most relevant. Additionally, accounting profit is most relevant 16 days after the financial statement submission deadline [31].

Considering these reasons, this study therefore hypothesizes that:

*H1a*: *Earnings per share have a positive effect on market value*.
*H1b*: *Book value per share has a positive effect on market value*.

### Financial activities and market value

#### Debt financing

Debt financing involves borrowing funds that a business must repay over time, either in the short term or long term. Short-term debt financing, typically due within a year, is used to address immediate financial needs, such as managing cash flow, financing inventory, or covering short-term liabilities; it includes instruments like lines of credit, commercial paper, and short-term bank loans. Long-term debt financing, with repayment periods extending beyond a year, is utilized for substantial investments like purchasing property, equipment, or business expansion and encompasses options like bonds, long-term bank loans, and mortgages. Short-term debt often features lower interest rates and quicker access to funds, while long-term debt generally involves higher interest rates due to prolonged risk exposure and necessitates a careful evaluation of the borrower’s long-term financial stability.

Debt financing plays a crucial role in shaping a firm’s operational and strategic decisions. Past research highlights that debt ratios, including trade credit, short-term debt, and long-term debt, negatively impact firm performance by reducing profitability [32]. High debt ratios also raise agency costs and increase the risk of losing control, leading many SME owners and managers to rely more heavily on equity financing [8]. Furthermore, financial leverage, especially the debt-to-EBITDA ratio, hurts important performance indicators like return on equity, earnings per share, and Tobin’s Q. This shows how important decisions about a company’s capital structure are for its overall performance [33]. Conversely, increases in both short-term and long-term debt, alongside tangible fixed assets, positively correlate with return on total assets, although long-term debt specifically reduces return on equity [34]. Past research also indicates that higher leverage ratios can enhance performance, particularly by improving return on equity due to tax benefits from interest expense deductions [35]. In developing economies, long-term debt financing has been found to mitigate liquidity imbalances caused by economic volatility, proving beneficial for firms in such environments [36]. Furthermore, significant debt financing has been associated with increased systematic risk [37] and negatively affects shareholder value and corporate investment, suggesting that excessive leverage may limit a firm’s ability to generate shareholder value and invest effectively [38]. Short-term debt positively influences financial growth, as evidenced by increased earnings per share and market capitalization [39]. However, raising short-term debt for long-term investment projects can significantly increase crash risk [40]. Replacing long-term debt with short-term debt may enhance expected stock returns by shifting risk from long-term debtholders to shareholders [41]. Moreover, long-term leverage obligations negatively impact firm value, though this effect varies based on firm size and the nature of long-term investments [42]. While the short-term debt to total assets ratio has little effect on firm value, long-term debt to total assets, total debt to total assets, and total debt-to-equity ratios positively affect firm value by reducing agency costs and information asymmetry while boosting investor confidence [43]. However, both short- and long-term debt can hinder profitability through agency issues that lead to high-debt policies, ultimately reducing overall firm performance [44].

Considering these reasons, this study therefore hypothesizes that:

*H2a*: *Short-term debt financing has a negative effect on market value*.
*H2b*: *Long-term debt financing has a negative effect on market value*.

#### Asset investments

Asset investments consist of current and fixed assets, which serve distinct roles. Current asset investments focus on assets that are quickly convertible into cash within a year, such as inventory, receivables, and cash itself, which are crucial for maintaining liquidity and financing day-to-day operations, while fixed asset investments focus on allocating capital to long-term assets like machinery, buildings, technology, and intangible assets, which are fundamental for enhancing production capacity, operational efficiency, and driving long-term growth. Both types of investments are essential because they contribute to strategic development and the competitive advantage of firms.

Past research indicates that research and development (R&D) investments have a positive effect on firm performance, albeit nonlinear. R&D investments increase firm value up to a certain point, beyond which additional investments yield diminishing returns [45]. This relationship is characterized by an inverted U-shaped curve, particularly pronounced in high-growth firms [46], suggesting the existence of an optimal investment level that maximizes firm value. This optimal level varies depending on the quality of investment opportunities and the presence of under- or over-investment issues [47]. Investments in marketing and brand capital are crucial for firm value enhancement. Marketing investments positively impact firm valuation [48]. Firms with lower brand capital investment generally achieve higher average stock returns compared to those with higher brand capital intensity, suggesting a positive correlation between brand capital and financial returns [49]. Investment efficiency plays a significant role in firm value, emphasizing the importance of effective resource allocation [50]. Additionally, the cumulative value of intangible assets, including computerized information, innovative property, and economic competence, positively influences sustainable growth rates and overall firm value [51]. Growth in short-term assets better predicts stock returns one year ahead, whereas long-term asset growth predicts stock returns two years in advance [52]. Furthermore, human assets positively relate to firm value, while fixed assets show a negative relationship. Efficiency in human resources has improved compared to the previous year, while efficiency in other assets has declined [53]. Fixed asset growth positively affects the sustainable growth rate, and this rate strengthens the impact of fixed asset growth on firm value [54]. Investments in fixed assets significantly impact profitability in the Nigerian banking sector [55]. However, investment in tangible assets negatively affects short-term returns, reflecting a profit-taking orientation among Indonesian capital market investors [56]. Lastly, changes in property, plant, and equipment drive the predictability of share returns with respect to non-current operating assets [57].

Considering these reasons, this study therefore hypothesizes that:

*H3a*: *Current asset investment has a positive effect on market value*.
*H3b*: *Fixed asset investment has a positive effect on market value*.

#### Dividend payout

The literature on corporate finance reveals divergent theories regarding the impact of dividend policies on firm valuation and investor behavior. According to signaling theory [58], investors utilize available information to make trading decisions, with executives typically possessing more information than external investors. This asymmetry of information leads executives to use dividends as a signaling tool to convey positive expectations about the firm’s future profitability, thereby building investor trust and potentially boosting stock prices [59]. The bird-in-hand theory, proposed by Lintner [60] and Gordon [61], posits that investors value immediate dividends over future, uncertain returns from stock price appreciation. This theory suggests that dividends are crucial as they provide direct and tangible returns to shareholders, thereby enhancing firm value. However, the Modigliani and Miller theorem [62] introduces a counterpoint to these perspectives by arguing that dividend policy is irrelevant to stock prices in perfect markets. According to MM theory, the intrinsic value of a firm is determined by its profitability and investment policies, not by how earnings are distributed between dividends and retained earnings. This perspective implies that rational investors are indifferent to the firm’s dividend policy, focusing instead on the firm’s overall profitability and future growth potential. These contrasting theories highlight the complexity of financial signaling and investor preferences, suggesting that the relevance and impact of dividend policies may vary depending on market conditions, firm-specific factors, and investor sentiments.

Past studies have extensively examined the impact of dividend policies on stock price dynamics, revealing nuanced relationships between dividend metrics and stock market behavior. A positive correlation between dividend yield and stock price changes has been observed, while a negative association exists between the dividend payout ratio and stock price fluctuations [63]. This finding is supported by the significant stock price increases that follow dividend announcements [10]. Furthermore, dividend policy has been demonstrated to serve as a reliable indicator of stock price volatility for industrial product firms in Malaysia [64]. Variations in dividend payments also influence stock trading values [65], with lower dividend yields correlating with heightened shareholder risk [66]. Past studies have also identified negative relationships between dividend payments, stock liquidity [67], and volatility [68]. Conversely, the influence of dividend policies on market speculation reveals significant impacts on speculative behaviors in the S&P 500 [69] and Gulf Cooperation Council firms [70]. Additionally, higher dividend payouts have been associated with reduced stock price risk [71]. Lastly, abnormal returns following dividend cutbacks suggest heightened market sensitivity to such announcements, highlighting the complex interplay between dividend signaling and market liquidity dynamics [72].

Considering these reasons, this study therefore hypothesizes that:

*H4*: *Dividend payout ratio has a positive effect on market value*.

### Sustainable development and market value

Past research underscores the intricate relationship between sustainable development practices and market value. It is common for companies on the ESG Index to have higher firm values [73]. Strong ESG ratings are linked to better financial and market performance [74], which shows how economic and social impacts are linked and how important ESG factors are [75]. In emerging economies, ESG practices correlate with financial ratios, highlighting the role of sustainability in shaping market outcomes [76]. Additionally, strong ESG performance is associated with reduced stock price volatility, suggesting its potential to mitigate risk [77]. ESG ratings are crucial in determining stock prices, particularly for insurance firms [78]. Enhancements in ESG performance can increase a company’s market value, with financial performance serving as a key mediating factor. Operational capacity also plays a significant role in mediating the relationship between ESG performance and market value [79]. The mechanisms through which environmental disclosure affects stock prices further underscore its importance in improving market transparency and governance [80]. Furthermore, ESG performance impacts investor confidence and management behavior [81]. A comprehensive evaluation of ESG factors is also crucial for reducing firm risks, enhancing investor trust, and promoting financial transparency and reliability [82].

Firms that adopt corporate sustainability practices have demonstrated superior stock market performance, even amidst challenges such as the COVID-19 pandemic [83]. Reporting on environmental sustainability positively impacts firm value, as increased responsibility and transparency, along with improved stakeholder trust, contribute to enhanced firm value [84]. Companies that disclose more environmental goals are more likely to secure membership in the Egyptian Sustainability Index and attain high sustainability rankings, which positively affects capital market reactions [85]. Sustainable development is positively associated with firms’ financial performance, particularly when measured by market capitalization [86]. Furthermore, firms that integrate sustainability issues into their operations generally leverage resources more effectively, resulting in stronger financial performance and greater shareholder value creation compared to their peers [87]. Additionally, companies with consistent green rankings for enhancing environmental performance exhibit significantly higher standardized cumulative abnormal returns compared to those with decreased or unchanged environmental performance. The environmental impact score, reflecting damage from operational activities, is also a critical factor in improving firm value [88]. Better sustainability performers are likely to positively influence firm profitability both in the immediate and subsequent periods [89]. Finally, firms that operate in alignment with ethical norms are valued by European investors, though results also reveal variability among markets, including periods before and after the global financial crisis [90].

Considering these reasons, this study therefore hypothesizes that:

*H5*: *Sustainable development enhances the relevance of accounting information and financial activities on market value*.

**Fig 1:**
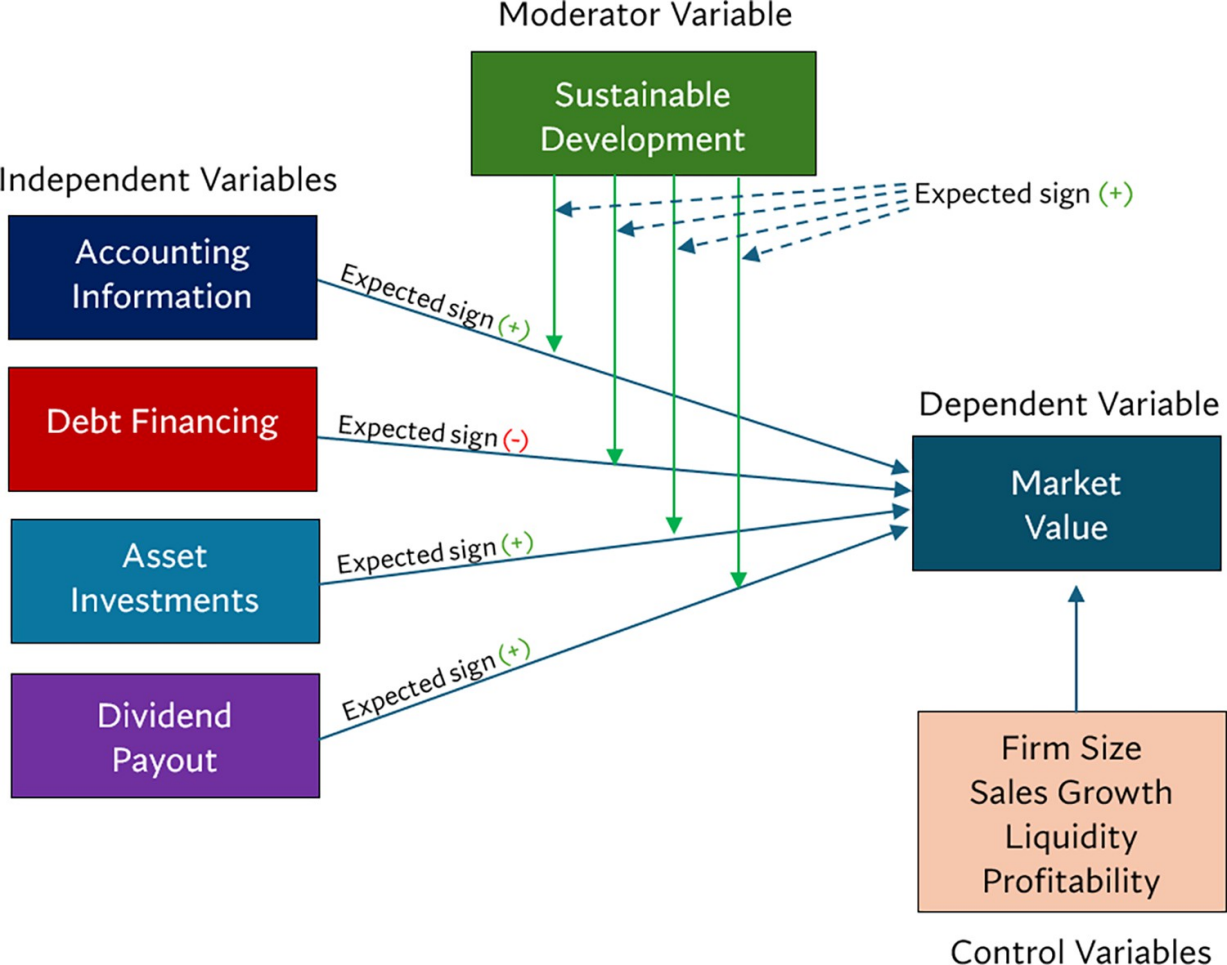
Research conceptual framework.

Based on the literature review, this study developed a research conceptual framework, depicted in Fig 1.

The research conceptual framework posits that sustainable development moderates the relationship between various financial factors and market value. Specifically, it suggests that, in the overall market, accounting information, asset investment, and dividend payout will have a positive impact on market value, while debt financing will have a negative effect. However, the strength of this relationship is changed by the sustainable development practices of the firm. It is expected that companies with a stronger commitment to sustainability are likely to experience a more pronounced positive relationship between these financial factors and their market value, including debt financing. Conversely, firms with lower levels of sustainable development may exhibit a weaker or even negative relationship. Additionally, the framework accounts for the influence of control variables such as firm size, sales growth, liquidity, and profitability, which may also affect market value.

## Methodology

This study analyzes the impact of sustainable development on the relevance of accounting information and financial activities. The research sample comprises companies listed on the Stock Exchange of Thailand from 2021 to 2023, with fiscal years ending on December 31^st^, and without a trading suspension exceeding 90 days. The sample also excludes firms within the financial sector and those that provide incomplete information. Multiple regression is employed to test the hypotheses.

Table 1 presents the definitions of the variables utilized in this study. The research hypothesis is tested through a model that uses market value per share (*MVPSt*_*+1*_) as the dependent variable, with accounting information and financial activities as independent variables. Indicators of accounting information include earnings per share (*EPS*_*it*_) and book value per share (*BVPS*_*it*_) [5], while financial activities encompass debt financing, asset investment, and dividend payments. Short-term debt financing (*SDEBT*_*it*_) and long-term debt financing (*LDEBT*_*it*_) serve as indicators of debt financing [43], while current asset investment (*CUR*_*it*_) [52] and fixed asset investment (*FIXED*_*it*_) [55] represent asset investment. The dividend payout ratio (*DP*_*it*_) is employed to measure firm dividend payouts [63]. Control variables include firm size (*SIZE*_*it*_), sales growth (*SG*_*it*_), liquidity (*LIQ*_*it*_), and profitability (*EBIT*_*it*_). Firm size is considered a control variable because larger companies have greater access to resources, which enhances performance, indicating a positive effect of firm size on firm value [91]. Sales growth is included to account for the company’s ability to expand its revenue base, providing insights for stakeholders, including investors and creditors, regarding future prospects; increases in sales without proportional costs suggest effective operational management, potentially leading to enhanced firm value [92]. Liquidity reflects the firm’s capacity to meet short-term obligations and has been shown to positively influence stock prices [93]. Lastly, profitability measures the firm’s efficiency in generating returns, which is critical for market valuation, with variability in profitability being a significant factor in explaining changes in company value [94]. Using these variables will ensure that the results reflect the relationship between the key factors under study, leading to more accurate outcomes. Model 1 can be expressed as follows:

$$MVPS_{it+1}=\beta_{0}+\beta_{1}EPS_{it}+\beta_{2}BVPS_{it}+\beta_{3}SDEBT_{it}+\beta_{4}LDEBT_{it}+\beta_{5}CUR_{it}+\beta_{6}FIXED_{it}+\beta_{7}DP_{it}+\beta_{8}SIZE_{it}+\beta_{9}SG_{it}+\beta_{10}LIQ_{it}+\beta_{11}EBIT_{it}+Industry\;Dummies+\varepsilon_{it}$$

where, *MVPS*_*it+1*_ denotes the market value per share of firm *i* in year *t+1*, utilizing the natural logarithm of market value per share on the date of the annual report publication. The independent and control variables incorporated in the model are defined in detail in Table 1. Here, *β*_0_ signifies the intercept term, *Industry Dummies* is the industry dummy variable, while *ε*_*it*_ represents the random error term.

**Table 1:** The variables definitions.

Symbols	Variables	Descriptions
**Panel A: Dependent variable**		
**MVPS_t+1_**	Market value per share	Natural logarithm of the market value per share on the date of the annual report publication.
**Panel B: Independent variables**		
**EPS_it_**	Earnings per share	Net income minus preferred dividends scaled by total common shares outstanding.
**BVPS_it_**	Book value per share	Shareholder’s equity minus preferred equity scaled by total common shares outstanding.
**SDEBT_it_**	Short-term debt financing	Short-term debt in year *t* scaled by total assets in year *t-1*.
**LDEBT_it_**	Long-term debt financing	Long-term debt in year *t* scaled by total assets in year *t-1*.
**CUR_it_**	Current asset investment	Current assets in year *t* scaled by total assets in year *t-1*.
**FIXED_it_**	Fixed asset investment	Fixed assets in year *t* scaled by total assets in year *t-1*.
**DP_it_**	Dividend payout ratio	Dividends per share scaled by earnings per share.
**Panel C: Moderator variable**		
**SD_it_**	Sustainable development	1 if the firm is in the SETESG Index and 0 otherwise.
**Panel D: Control variables**		
**SIZE_it_**	Firm size	Natural logarithm of total assets.
**SG_it_**	Sales growth	Sales in year *t* minus sales in year *t-1*, scaled by the sales in year *t-1* and multiplied by 100.
**LIQ_it_**	Liquidity	Current asset scaled by current liabilities.
**EBIT_it_**	Profitability	Earnings before interest and tax scaled by revenue and multiplied by 100.

Sustainable development assessments exhibit considerable variation across countries, reflecting diverse approaches to evaluating corporate sustainability performance. For example, inclusion in the Dow Jones Sustainability Indices (DJSI) signifies a firm’s exceptional efforts in addressing ESG issues [95]. In Brazil, the São Paulo Stock Exchange introduced the Corporate Sustainability Index and the Carbon Efficient Index to evaluate firms’ sustainable performance [96]. Similarly, the Stock Exchange of Thailand established the Thailand Sustainability Investment (THSI) in 2015, later renamed SETESG in 2023. This index compiles stocks of firms committed to sustainable practices aligned with ESG principles, providing investors with opportunities to invest in firms demonstrating outstanding ESG performance and supporting sustainable business practices from social and environmental perspectives. Consequently, this research utilizes the inclusion in the SETESG index as an indicator of firms that adhere to sustainable development guidelines and establishes sustainable development (*SD*_*it*_) as a moderator variable, which will interact with the relationship between the independent variables (accounting information, debt financing, asset investment, and dividend payout) and the dependent variable (market value). The moderator variable modifies the strength or direction of the relationship between independent and dependent variables, investigating how the effects of accounting information and financial activities on market value are contingent upon the presence or emphasis of sustainable development practices. Model 2 can be expressed as follows:

$$MVPS_{it+1}=\beta_{0}+\beta_{1}EPS_{it}+\beta_{2}BVPS_{it}+\beta_{3}SDEBT_{it}+\beta_{4}LDEBT_{it}+\beta_{5}CUR_{it}+\beta_{6}FIXED_{it}+\beta_{7}DP_{it}+\beta_{8}(EPS_{it}\;x\;SD_{it})+\beta_{9}(BVPS_{it}\;x\;SD_{it})+\beta_{10}(SDEBT_{it}\;x\;SD_{it})+\beta_{11}(LDEBT_{it}\;x\;SD_{it})+\beta_{12}(CUR_{it}\;x\;SD_{it})+\beta_{13}(FIXED_{it}\;x\;SD_{it})+\beta_{14}(DP_{it}\;x\;SD_{it})+\beta_{15}SIZE_{it}+\beta_{16}SG_{it}+\beta_{17}LIQ_{it}+\beta_{18}EBIT_{it}+Industry\;Dummies+\varepsilon_{it}$$

where, *MVPS*_*it+1*_ denotes the market value per share of firm *i* in year *t+1*, utilizing the natural logarithm of market value per share on the date of the annual report publication. *SD*_*it*_ represents sustainable development, using dummy variables, with a value of 1 if the firm is included in the SETESG Index and 0 otherwise. The independent and control variables incorporated in the model are defined in detail in Table 1. Here, *β*_0_ signifies the intercept term, *Industry Dummies* is the industry dummy variable, while *ε*_*it*_ represents the random error term.

## Results and discussion

### Descriptive statistics

Table 2 provides descriptive statistics for the variables under examination. The mean market value per share is 1.696, with a standard deviation of 1.562. Earnings per share exhibit a mean value of 0.947 and a standard deviation of 3.118. The mean book value per share is 12.436, accompanied by a standard deviation of 34.000. Short-term debt financing has a mean value of 0.282 and a standard deviation of 0.193, while long-term debt financing has a mean value of 0.198 and a standard deviation of 0.230. Current asset investment has a mean value of 0.467 with a standard deviation of 0.276, whereas fixed asset investment exhibits a mean value of 0.622 with a standard deviation of 0.383. The dividend payout ratio has a mean value of 0.306 and a standard deviation of 0.653. The firm size is characterised by a mean value of 8.978 and a standard deviation of 1.596. Sales growth has a mean value of 22.389 and a standard deviation of 101.164. Liquidity has a mean value of 2.774, accompanied by a standard deviation of 5.127, while profitability exhibits a mean value of 58.561 and a standard deviation of 1380.606.

**Table 2:** Descriptive statistics.

Variables	N	Minimum	Maximum	Mean	SD.
**MVPS_t+1_**	1401	-4.605	6.207	1.696	1.562
**EPS_it_**	1401	-41.110	39.310	0.947	3.118
**BVPS_it_**	1401	-0.450	490.720	12.436	34.000
**SDEBT_it_**	1401	0.001	1.600	0.282	0.193
**LDEBT_it_**	1401	0.000	4.870	0.198	0.230
**CUR_it_**	1401	-0.544	2.499	0.467	0.276
**FIXED_it_**	1401	0.020	8.080	0.622	0.383
**DP_it_**	1401	0.000	14.430	0.306	0.653
**SIZE_it_**	1401	5.411	15.057	8.978	1.596
**SG_it_**	1401	-100.000	1793.990	22.389	101.164
**LIQ_it_**	1401	-34.930	77.120	2.774	5.127
**EBIT_it_**	1401	-9632.980	44706.280	58.561	1380.606

### Regression analysis

The Pearson correlation matrix and variance inflation factor (VIF) results for the variables are shown in Table 3. These show several significant relationships that need to be looked at in more detail. For instance, the robust positive correlation of 0.473 between earnings per share and book value per share suggests that higher earnings are associated with increased asset valuations, reflecting a potential alignment between a firm’s profitability and intrinsic value. Additionally, the positive correlation between fixed asset investment and long-term debt financing, recorded at 0.737, indicates that firms with substantial investments in fixed assets often rely heavily on long-term financing to facilitate capital expenditures. The current asset investment also exhibits a significant positive correlation of 0.433 with short-term debt financing, implying that firms with stronger current asset investment positions may strategically utilize more short-term debt to optimize their investment. Moreover, the positive correlation of 0.221 between firm size and fixed asset investment suggests that larger firms tend to engage in substantial capital investments. Sales growth, with a positive correlation of 0.290 with long-term debt financing, indicates that growing firms may increasingly rely on long-term financing to support expansion efforts. Lastly, liquidity demonstrates a negative correlation of -0.313 with short-term debt financing, suggesting that firms with higher liquidity levels tend to maintain lower short-term debt obligations, favoring internal financing mechanisms. Overall, the Pearson correlation matrix and VIF results don’t show any problems with multicollinearity between the variables. This is because all significant Pearson correlations stay below 0.80 [97], and VIF values are less than 10 [98]. This thorough assessment of multicollinearity, achieved by excluding multicollinear explanatory variables, strengthens the reliability of the multiple linear regression model [99]. Consequently, these findings support the validity of subsequent analyses involving these variables.

**Table 3:** Pearson correlations matrix and variance inflation factor.

Variables	MVPS_t+1_	EPS_it_	BVPS_it_	SDEBT_it_	LDEBT_it_	CUR_it_	FIXED_it_	DP_it_	SIZE_it_	SG_it_	LIQ_it_	EBIT_it_	VIF
**MVPS_t+1_**	1												
**EPS_it_**	0.406**	1											1.376
**BVPS_it_**	0.470**	0.473**	1										1.326
**SDEBT_it_**	-0.138**	-0.049	-0.095**	1									1.625
**LDEBT_it_**	0.088**	0.008	0.022	0.047	1								2.924
**CUR_it_**	0.027	0.034	-0.087**	0.433**	-0.204**	1							1.952
**FIXED_it_**	.071**	0.040	0.049	-0.007	0.737**	-0.425**	1						3.029
**DP_it_**	0.103**	0.021	-0.001	-0.094**	-0.079**	0.024	-0.085**	1					1.026
**SIZE_it_**	0.342**	0.223**	0.197**	0.052	0.423**	-0.191**	0.221**	0.024	1				1.503
**SG_it_**	0.035	-0.037	0.006	0.049	0.290**	-0.012	0.299**	-0.065*	-0.002	1			1.145
**LIQ_it_**	0.008	0.010	0.008	-0.313**	-0.181**	0.140**	-0.148**	0.052	-0.212**	-0.039	1		1.389
**EBIT_it_**	0.082**	0.042	0.001	-0.045	-0.026	-0.046	0.025	0.042	.054*	-0.045	0.260**	1	1.110

Note

**Correlation is significant at the 0.01 level (two-tailed)

*Correlation is significant at the 0.05 level (two-tailed)

The regression analysis presented in Table 4 reveals that earnings per share has a significant positive effect on market value per share (p < 0.001) with an adjusted R-squared value of 23.70%. This result is supported by the premise that higher earnings signal strong financial performance and profitability, thereby attracting investor interest. Furthermore, results find that book value per share has a positive effect on market value per share (p < 0.001) with an adjusted R-squared value of 29.10%, underscoring its importance in asset valuation as it reflects the underlying worth of a company’s assets relative to its equity, providing a basis for assessing worth. These results are consistent with studies conducted in Egypt, which found that earnings and book values hold substantial explanatory power for stock prices [4]. Similarly, studies in ASEAN banks [29] and Nigeria [23], which found that earnings per share and net book value per share positively influence share prices, suggest that earnings per share and book value per share significantly contribute to explaining the positive market value, implying that strong profitability drives higher market values.

**Table 4:** Predictive power of individual components.

Variables	MVPS_t+1_	MVPS_t+1_	MVPS_t+1_	MVPS_t+1_	MVPS_t+1_	MVPS_t+1_	MVPS_t+1_
**EPS_it_**	0.345*** (14.364)						
**BVPS_it_**		0.417*** (18.117)					
**SDEBT_it_**			-0.149*** (-5.706)				
**LDEBT_it_**				-0.081** (-2.772)			
**CUR_it_**					0.092*** (3.609)		
**FIXED_it_**						-0.012 (-0.431)	
**DP_it_**							0.093*** (3.709)
**SIZE_it_**	0.274*** (11.107)	0.267*** (11.267)	0.352*** (13.817)	0.388*** (13.702)	0.370*** (14.238)	0.357*** (13.582)	0.352*** (13.718)
**SG_it_**	0.053* (2.245)	0.038 (1.677)	0.046 (1.875)	0.064* (2.431)	0.042 (1.676)	0.044 (1.683)	0.047 (1.864)
**LIQ_it_**	0.054* (2.175)	0.048* (2.012)	0.024 (0.881)	0.067* (2.498)	0.062* (2.308)	0.072** (2.676)	0.068** (2.576)
**EBIT_it_**	0.041 (1.690)	0.056* (2.395)	0.052* (2.020)	0.045 (1.714)	0.052* (2.007)	0.046 (1.778)	0.044 (1.678)
**Industry**	Yes	Yes	Yes	Yes	Yes	Yes	Yes
**Obs.**	1401	1401	1401	1401	1401	1401	1401
**adj. R^2^**	23.70%	29.10%	14.40%	12.90%	13.20%	12.40%	13.30%

**Note:** The t-statistics are calculated and reported in parentheses

*** p < 0.001

** p < 0.01, and

* p < 0.05, respectively.

Short-term debt financing has a negative impact on market value per share (p < 0.001), with an adjusted R-squared value of 14.40%, implying that investors have concerns about increasing short-term debt financing will lead to increased future financial risk and potential cash flow issues. Furthermore, long-term debt financing has a significantly negative effect on market value per share (p < 0.01), with an adjusted R-squared value of 12.90%. Although the long-term debt financing effect is less pronounced than that of short-term debt financing, it reflects concerns about long-term financial obligations that may affect future profitability too. These results align with the past studies indicating that debt ratios, including trade credit, short-term debt, and long-term debt, have a negative effect on firm performance [32], increase systematic risk [37], and adversely affect shareholder value and firm investment [38]. The adjusted R-squared value shows that, while debt financing metrics have a statistically significant negative effect on market value, they explain a smaller portion of the variability of market value compared to earnings per share and book value per share. This might be because debt financing is complicated by outside factors like interest rates, and market conditions also impact firm market value, making debt financing less powerful for explaining the market value.

The current asset investment has a positive effect on market value per share (p < 0.001), with an adjusted R-squared of 13.20%, indicating that investors value firms with sufficient current assets, as they reflect firm ability to meet short-term obligations and manage operational needs effectively. The positive impact implies that well-managed current assets can improve investor confidence, thereby increasing the overall market value of the firm. In contrast, fixed asset investment has a negligible negative effect on market value per share, which is not statistically significant (p > 0.05), with an adjusted R-squared of 12.40%, indicating that investors prioritize cash flows over fixed assets in their valuation processes. The results align with past studies that found growth in short-term assets better predicts stock returns one year ahead than long-term assets [52]. However, these results contrast with earlier research indicating that fixed asset growth positively affects the sustainable growth rate, which in turn strengthens the impact of fixed asset growth on firm value [54]. The adjusted R-squared values indicate their effect on explaining market value. While these values suggest that both types of investment have impacts, other elements not captured in the model may also significantly influence market valuation. This could indicate that asset investment does not always correlate with market perceptions of value, possibly due to other factors such as asset depreciation or market volatility.

The dividend payout ratio has a significantly positive effect on market value (p < 0.001), with an adjusted R-squared of 13.30%, indicating that dividend payments are valued by investors as a signal of financial health and shareholder returns. The result aligns with the bird-in-hand theory, which posits that dividends are crucial because they provide direct and tangible returns to shareholders, thereby enhancing firm value, and is consistent with past studies indicating a positive correlation between dividend yield and stock price changes [63], as well as findings that stock prices tend to increase following dividend announcements [10]. Although a higher adjusted R-squared value would suggest a stronger relationship, the 13.30% figure indicates that other factors play a significant role in determining market value too. This impact may be attributed to investor preferences for dividends as signals of firm financial health and profitability. However, it also suggests that overall market value is influenced by a range of factors beyond just dividend distributions. Overall, the adjusted R-squared values in this analysis illustrate the varying degrees to which different financial metrics describe market value per share, underscoring the importance of carefully selecting variables that truly reflect the complexities of market dynamics.

In terms of control variables, firm size consistently has a positive effect on market value per share across models (p < 0.001), indicating that larger firms will achieve higher market valuations from investors, likely due to their competitive advantages and firm stability. Also, both sales growth and firm liquidity have positive effects. Sales growth has mixed significance, but liquidity has a positive effect on several models. This suggests that the ability to grow sales and manage liquidity well is important for keeping investors’ confidence. Furthermore, profitability demonstrates a positive effect but a less pronounced impact, indicating that while profitability is an essential indicator of firm financial performance, its influence is often overshadowed by clearer metrics like earnings per share or dividend payouts. Overall, while several factors contribute to market value, earnings per share and book value per share emerge as the most influential indicators.

Table 5 illustrates the relevance of accounting information and financial activity metrics. The results indicate that the relevance of earnings per share and book value per share positively affects market value per share (p < 0.001), with an adjusted R-squared of 32.00%. Additionally, the analysis of the relevance of accounting information and short-term debt financing finds that both earnings per share and book value per share have a positive effect on market value per share (p < 0.001), while short-term debt financing has negative effects (p < 0.001), with an adjusted R-squared of 33.00%. Similarly, the analysis of the relevance of accounting information and long-term debt financing finds that accounting information positively affects market value per share (p < 0.001), whereas long-term debt financing does not exhibit statistical significance (p > 0.05), with an adjusted R-squared of 32.00%. Moreover, the analysis of the relevance of accounting information and current asset investment reveals that accounting information positively affects market value per share (p < 0.001), and current asset investment also has a positive effect at the same level of significance, with an adjusted R-squared of 32.80%. Furthermore, the result of the relevance of accounting information and fixed asset investment suggests that accounting information positively influences market value per share (p < 0.001), while fixed asset investment does not demonstrate statistical significance (p > 0.05), with an adjusted R-squared of 31.90%. Finally, the analysis of the relevance of accounting information and dividend payout ratio demonstrates that accounting information positively affects market value per share (p < 0.001), and the dividend payout ratio also has a positive effect at the same level of significance, with an adjusted R-squared of 32.80%. The adjusted R-squared values for each model in this table indicate how well each model explains the relevance of accounting information and financial activity metrics on market value. The model includes short-term debt financing, which has the highest adjusted R-squared value (33%). This means that accounting information and short-term debt financing together explain a larger part of the variation in market value. Furthermore, the model for current asset investments follows closely, with an adjusted R-squared value of 32.80%, indicating that current asset investments positively contribute to market value when combined with accounting information. The models incorporating only accounting information metrics, as well as models including long-term debt financing and the dividend payout ratio, show similar explanatory power, with adjusted R-squared values ranging from 32.00% to 32.80%, indicating moderate relevance. Lastly, the adjusted R-squared value for fixed asset investment is the lowest, at 31.90%, possibly because investors rarely place significant importance on firm fixed assets. Therefore, these research results indicate that the value relevance of accounting information and short-term debt financing demonstrates the greatest explanatory power on market value, whereas fixed asset investment has the least explanatory power.

**Table 5:** The value relevance of accounting information and financial activity metrics.

Variables	MVPS_t+1_	MVPS_t+1_	MVPS_t+1_	MVPS_t+1_	MVPS_t+1_	MVPS_t+1_
**EPS_it_**	0.196*** (7.749)	0.196*** (7.791)	0.194*** (7.656)	0.184*** (7.251)	0.197*** (7.749)	0.195*** (7.731)
**BVPS_it_**	0.329*** (13.067)	0.319*** (12.727)	0.328*** (13.012)	0.341*** (13.531)	0.329*** (13.071)	0.331*** (13.202)
**SDEBT_it_**		-0.108*** (-4.664)				
**LDEBT_it_**			-0.035 (-1.370)			
**CUR_it_**				0.098*** (4.342)		
**FIXED_it_**					-0.017 (-0.704)	
**DP_it_**						0.093*** (4.234)
**SIZE_it_**	0.239*** (10.200)	0.239*** (10.277)	0.255*** (9.810)	0.256*** (10.829)	0.243*** (10.143)	0.236*** (10.130)
**SG_it_**	0.045* (2.041)	0.049* (2.244)	0.055* (2.371)	0.046* (2.078)	0.050* (2.158)	0.051* (2.308)
**LIQ_it_**	0.043 (1.817)	0.008 (0.327)	0.040 (1.701)	0.031 (1.302)	0.041 (1.728)	0.038 (1.628)
**EBIT_it_**	0.051* (2.234)	0.056* (2.440)	0.051* (2.211)	0.059* (2.562)	0.052* (2.271)	0.049* (2.152)
**Industry**	Yes	Yes	Yes	Yes	Yes	Yes
**Obs.**	1401	1401	1401	1401	1401	1401
**adj. R^2^**	32.00%	33.00%	32.00%	32.80%	31.90%	32.80%

**Note:** The t-statistics are calculated and reported in parentheses

*** p < 0.001

** p < 0.01, and

* p < 0.05, respectively.

The analysis in Table 6 provides the value relevance of accounting information and financial activities and the moderating effect of sustainable development. The results indicate that, in the overall market, earnings per share and book value per share significantly positively influence market value (p < 0.001), indicating that rising profits align with increased market value and highlighting the positive impact of financial performance indicators on market valuation, aligning with the past study [5], which found that earnings and book value have a statistically significant relationship with firm value. Conversely, debt financing significantly negatively affects market value (p < 0.001) due to heightened financial risk and reduced flexibility; elevated debt levels result in higher interest payments, which can erode profits and amplify financial vulnerability, consistent with the past study [44], which found that both short- and long-term debt financing adversely impact firm profitability due to potential agency issues fostering high-debt policies, ultimately hampering performance. Furthermore, asset investment has a significantly positive effect on market value (p < 0.001) by signaling confidence in future growth and operation performance, leading to increased market valuation through improved earnings and cash flows, consistent with the past study [52], which indicates that asset growth has a significant ability to anticipate stock returns. Also, the dividend payout ratio has a significantly positive effect on market value (p < 0.001), which indicates excellent financial health and profit stability. Firms that distribute dividends demonstrate a commitment to returning value to investors, leading to enhanced firm reputation and investor perception, consistent with the past study [63], which found a positive correlation between dividend yield and stock price changes. These results suggest that investors view these metrics as reliable indicators of financial health, reflecting strong explanatory power to market value with an adjusted R-squared of 37.20%.

**Table 6:** The value relevance of accounting information and financial activities, and the moderating effect of sustainable development.

Variables	MVPS_t+1_	MVPS_t+1_
**EPS_it_**	0.153*** (6.167)	0.195*** (6.961)
**BVPS_it_**	0.334*** (13.691)	0.397*** (14.678)
**SDEBT_it_**	-0.234*** (-8.661)	-0.236*** (-8.624)
**LDEBT_it_**	-0.119*** (-3.278)	-0.106** (-2.810)
**CUR_it_**	0.265*** (8.964)	0.279*** (9.245)
**FIXED_it_**	0.163*** (4.416)	0.144*** (3.861)
**DP_it_**	0.073*** (3.386)	0.056* (2.475)
**EPS_it_ x SD_it_**		-0.071* (-2.009)
**BVPS_it_ x SD_it_**		-0.117*** (-3.313)
**SDEBT_it_ x SD_it_**		0.148** (2.883)
**LDEBT_it_ x SD_it_**		-0.057 (-1.023)
**CUR_it_ x SD_it_**		-0.052 (-1.195)
**FIXED_it_ x SD_it_**		0.175** (2.773)
**DP_it_ x SD_it_**		-0.002 (-0.087)
**SIZE_it_**	0.301*** (11.590)	0.238*** (8.157)
**SG_it_**	0.045* (1.997)	0.045* (2.032)
**LIQ_it_**	-0.059* (-2.383)	-0.058* (-2.372)
**EBIT_it_**	0.068* (3.045)	0.060* (2.652)
**Industry**	Yes	Yes
**Obs.**	1401	1401
**adj. R^2^**	37.20%	40.60%

**Note:** The t-statistics are calculated and reported in parentheses

*** p < 0.001

** p < 0.01, and

* p < 0.05, respectively.

In the context of firms following sustainable development guidelines, earnings per share and book value per share have significantly negative effects on market value (p < 0.05 and p < 0.001, respectively), which indicates that investors perceive sustainability as a cost rather than a benefit. High earnings per share and book value per share as only temporary gains overshadowed by the perceived financial burdens of sustainability efforts suggest that even strong earnings per share and book value per share figures do not fully offset the concern of substantial capital investments for sustainability initiatives. This belief leads to concerns that such firms may face future earnings challenges, particularly if sustainability initiatives fail to yield financial returns commensurate when compared with the investment capital the firm has invested, leading to the current market valuation being lower. Conversely, short-term debt financing has a significantly positive effect on market value (p < 0.01), suggesting that while sustainability initiatives incur upfront costs, short-term debt can provide a flexible and efficient way to fund these investments without diluting equity or incurring long-term debt obligations, helping maintain a strong financial position and reduce future financial distress. However, long-term debt financing shows a non-significant negative effect on market value (p > 0.05), indicating that sustainability initiatives often require substantial upfront capital expenditures, which may take time to generate returns. When coupled with long-term debt, this creates a dual financial burden on the firm: ongoing interest payments on the debt and the investment cost for sustainability projects. However, the impact of long-term debt financing on investors may be less pronounced when compared to other factors, such as broader economic conditions and prevailing industry trends, which alleviate concerns about long-term debt. Current asset investment has an insignificantly negative effect on market value (p > 0.05), suggesting that current asset investments, when allocated to sustainability initiatives, tend to tie up capital without generating immediate returns. Investors are concerned that the firm is diverting essential resources away from more profitable ventures, thereby reducing profitability. This perspective can lead investors to undervalue the stock. However, investment in current assets may not clearly reflect operating performance when compared to other indicators, such as earnings per share and book value per share, and thus have an insignificant impact on market value. Fixed asset investments have a significant positive effect on market value (p < 0.01), indicating that while the upfront costs may raise concerns about short-term profitability, these investments typically lead to enhanced operational efficiencies, reduced energy costs, and lower regulatory risks. Over time, firms that integrate sustainability into their fixed assets, such as renewable energy systems or energy-efficient facilities, experience improved cash flow and competitive positioning; in the worst-case scenario, if sustainability investments fail, fixed assets, such as critical infrastructure, can still be leveraged to improve operational efficiency. This long-term perspective gradually shifts investor confidence, enhancing the market valuation. Finally, the dividend payout ratio has an insignificantly negative effect on market value (p > 0.05), indicating that a high dividend payout diverts capital away from sustainable development initiatives. Consequently, firms need to allocate additional capital to these initiatives, which worsens the existing financial constraints. However, various factors, such as the broader economic and industry context, may also influence the overall impact on market value, resulting in an insignificant impact.

These results are consistent with the efficient market theory [18], which posits that stock markets respond quickly to news, reflecting investor concerns about economic impacts. Concerns regarding investments in sustainability projects may impose a significant financial burden on the company. Firm value will decrease if these initiatives fail to generate profitable returns [16]. This research results challenge the notion that sustainable development is positively associated with financial performance, particularly when measured by market capitalization [86]. Although past research has found that firms integrating sustainability into their operations generally leverage resources more effectively, resulting in stronger financial performance and greater shareholder value creation [87], this study findings suggest otherwise. When weighing the potential benefits—such as an enhanced corporate image—against the substantial capital expenditures required, investors might perceive such investments as unjustifiable. Consequently, even if a company demonstrates positive current performance, investors may respond negatively to the financial information presented. In model 2, the adjusted R-squared of 40.60% indicates that the value relevance of accounting information and financial activities of firms adhering to sustainable development guidelines has a significant influence on market value because of the concern of substantial upfront costs typically incur when implementing sustainable investment, which investors perceive as detrimental to future profitability and cash flow. Such cautious perceptions lead to a more negative investor response to sustainability disclosures, overshadowing other financial metrics, resulting in a disproportionately negative impact on the market valuation of these firms.

## Conclusion

In the overall market, earnings per share and book value per share are critical market value drivers, with consistent earnings growth enhancing firm value. However, debt financing undermines firm profitability due to increased financial risk and interest obligations, resulting in a decrease in market value. Asset investments are signs of firm growth, enhancing market valuation through improved operational efficiency and innovation, while dividend payouts bolster market value by indicating financial health and commitment to returning value to shareholders. However, in the context of firms adhering to sustainable development guidelines, they encounter more complex dynamics. While they report high earnings per share and book value per share, these financial metrics cannot alleviate investor skepticism regarding sustainability as a cost of the firm. The substantial investments for sustainability initiatives continue to raise concerns about future firm profitability, leading to a negative impact on market value. Furthermore, short-term debt financing offers a favorable avenue for funding these initiatives, while the implications of long-term debt financing and current asset investments remain ambiguous. Furthermore, the significant positive effect of fixed asset investment underscores the potential long-term benefits of sustainability, despite high initial costs. Lastly, the non-significant negative impact of dividend payouts on market value suggests that the overall effect may also depend on various factors. Based on these findings, this study recommends that policymakers should carefully design regulations and incentives to support sustainable investments. Governments may consider establishing specific funds to support sustainability projects, providing tax incentives, grants, or low-interest financing options to alleviate financial burdens on firms investing in sustainable practices. Additionally, it is crucial to encourage transparency and consistent reporting on the long-term financial benefits of sustainability through an annual sustainability report that more details their sustainability initiatives, objectives, and progress. This report should include measurable outcomes, such as reductions in carbon emissions, energy savings, and improvements in operational efficiency, and it must be able to indicate a trend toward increased financial performance. This approach could help reduce investor skepticism and foster a more positive market response. Furthermore, firms should recognize that sustainability initiatives need to be balanced with financial performance to maintain investor confidence. It is essential for companies to effectively communicate the potential long-term benefits of these investments, demonstrating how sustainable practices contribute to operational efficiency, cost savings, and risk mitigation. Moreover, adopting short-term financing options may help firms manage immediate costs without compromising their financial stability. Strategic investments in fixed assets that align with sustainability goals should be prioritized, as these are more likely to yield positive market responses in the long run. Ultimately, the findings challenge the traditional view that sustainable development is always positively correlated with financial performance. Policymakers and firms must recognize the nuanced relationship between sustainability efforts and market valuation to develop strategies that effectively address investor concerns.

Future research should explore several avenues, such as longitudinal studies, which could provide insights into the evolving nature of sustainability initiatives and their impact on financial performance over time. Looking into how firm-specific factors, industry dynamics, and outside factors like regulatory frameworks and market sentiment affect the relationship between sustainability and financial performance could help us understand it better. Comparative studies of different regions or industries could also show differences in how well sustainability strategies work and what that means for the usefulness of accounting information and financial activities in various situations. Lastly, exploring the sentiment of diverse stakeholders, including investors, regulators, and consumers, toward sustainable development initiatives could offer valuable insights for firms aiming to align their strategies with stakeholder expectations and market demands.
